# Trochlear Osteonecrosis After a Nonoperative Lateral Humeral Condyle Fracture in a Child

**DOI:** 10.5435/JAAOSGlobal-D-19-00101

**Published:** 2020-05-01

**Authors:** David Alex Hamilton, Kunal Kalra

**Affiliations:** From the Department of Orthopaedic Surgery, Detroit Medical Center, Children's Hospital of Michigan, Detroit, MI.

## Abstract

This patient originally presented at the age of 2 years with a 1-mm displaced lateral humeral condyle fracture after a fall. He was treated nonoperatively in a long-arm cast, and serial x-rays were followed for 1 month. At the 1-month clinic visit, the lateral condyle demonstrated excellent alignment and healing, and the long-arm cast was removed. He eased back into activities and was doing well at 3 months. He returned over 4 years later, at the age of 7 years, with the report of elbow clicking with motion. At that time, imaging demonstrated collapse of the ulnohumeral articulation.

Osteonecrosis of the trochlea, sometimes called Hegemann disease, is a rare condition of the distal humerus that is seen in children. A recently published literature review found a total of eight cases because it was first described in the 1950s.^1^ The pathology exists on a poorly-defined spectrum that also includes fishtail deformity and dissolution of the humeral trochlea. Taking this full spectrum of conditions into account, it is still an incredibly uncommon presentation.^1,2,3,4^ Both traumatic and atraumatic etiologies have been reported.^1^ In the traumatic cases, elbow contusions and elbow fractures have both been implicated in the development of Hegemann disease. The patient in our case report also has a history of a fracture with subsequent development of trochlear osteonecrosis.

This patient presented at 2 years old after a fall from a small height. X-rays demonstrated a 1-mm displaced lateral condyle fracture of his left humerus (Figure 1). The patient was treated nonoperatively in a long-arm cast as per the standard of care.^5^ Given the minimal displacement, a closed reduction maneuver was not deemed necessary. At his 1-month follow-up appointment, x-rays showed excellent healing and maintained alignment of the fracture (Figure 2), and his cast was removed. He eased back into activities and was doing well at 3 months postinjury, both clinically and on radiograph (Figure 3).

**Figure 1:**
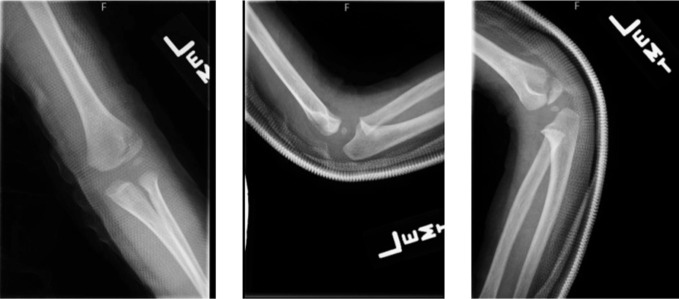
Radiographs demonstrating the AP, lateral, and oblique x-rays of the left elbow from when the patient first presented to clinic. Imaging shows an approximately 1-mm displaced lateral condyle fracture of the distal humerus.

**Figure 2:**
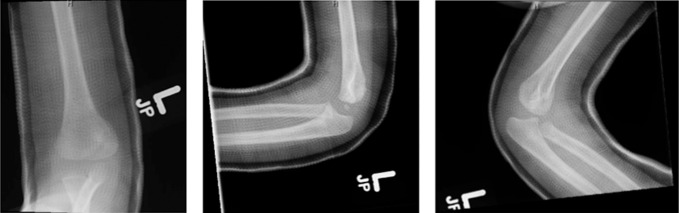
Radiographs demonstrating the AP, lateral, and oblique x-rays of the left elbow at the patient's 1-month follow-up visit. Images show maintained alignment and interval healing of the lateral condyle fracture.

**Figure 3:**
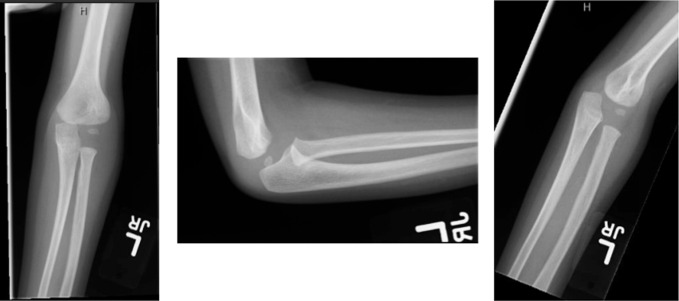
Radiographs demonstrating the AP, lateral, and oblique x-rays of the left elbow at the patient's 3-month follow-up visit. Images show maintained alignment and full healing of the lateral condyle fracture.

The patient returned 4 years later, at the age of 7 years, with the chief report of “elbow clicking.” He was not having any pain. However, x-rays at that visit showed poor congruence at the articular surface between the ulna and distal humerus (Figure 4). The patient obtained an MRI of the left elbow, as shown in Figures 5 and 6. He was found to have cartilaginous collapse of the left trochlea.

**Figure 4:**
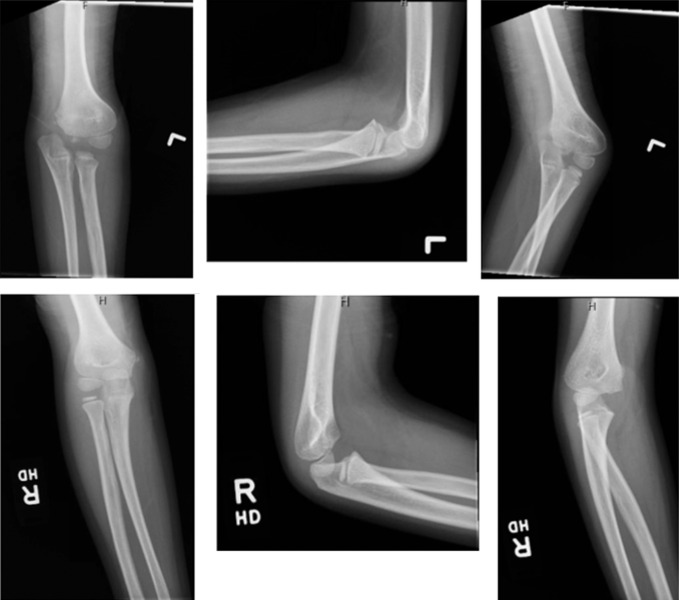
Radiographs demonstrating the AP, lateral, and oblique x-rays of the left and right (unaffected) elbows 4 years after the initial injury. The left elbow films show the lack of congruence at the ulnohumeral articulation because of the collapse of the articular cartilage.

**Figure 5:**
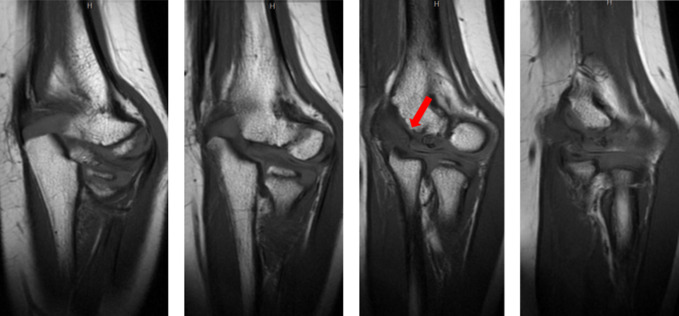
Radiographs demonstrating the sequential coronal T1-weighted MRI images of the left elbow. Breakdown of normal bony surface of the medical distal humerus shown (red arrow).

**Figure 6:**
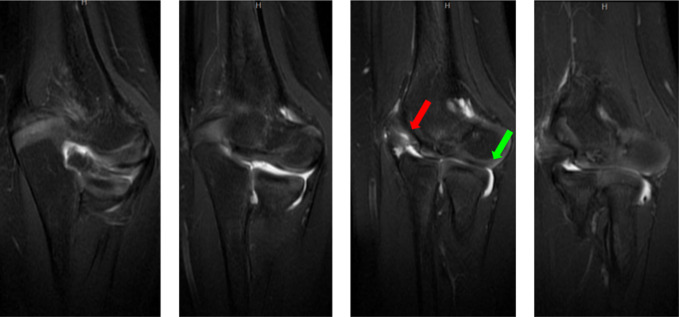
Radiographs demonstrating the sucessive coronal T2-weighted MRI images of the left elbow. Breakdown of normal cartilaginous surface of the trochlea shown (red arrow). For comparison, the normal cartilage of the capitellum is also demonstrated (green arrow).

The patient followed up in clinic again a couple months after the MRI. He and his mother had noticed progressive deformity about the elbow. He still had a full range of motion and no pain. We discussed the possibility of doing an elbow arthrogram to further elucidate the bony and cartilaginous anatomy. However, the family opted for continued monitoring, given he remained fully functional and had no pain. We also opted against a CT scan at this time because we did not think that it would change his treatment, and the results of the test were not worth the risk of radiation exposure. We also discussed that the patient might need future surgery about the elbow to correct deformity or improve function. The family is aware of this, and the plan is to continue monitoring.

## Discussion

The pediatric elbow develops in a stepwise and predictable fashion.^6^ The trochlea ossifies around 7 years of age and fuses at around 12 years. Before ossification, the trochlear ossification center is covered in articular cartilage. Osteonecrosis involves collapse of both bone and articular cartilage because of the lack of blood supply.^7^ The distal medial humerus and its ulnar articulation should have a congruent bony appearance because we can see in the bottom images of Figure 4 on the contralateral side of this patient. On our patient's affected side (top images of Figure 4), we can see what seems to be the collapse of the articular cartilage of the trochlea and poor articulation at the ulnohumeral joint.

The blood supply to the pediatric elbow is a complicated anastomotic network of vessels. An extraosseous and an intraosseous system supplies the area about the distal humerus. The extraosseous blood supply is supplied by the brachial artery anteriorly, with anastomotic branches that course posteriorly.^8^ The anatomy of the intraosseous blood supply might help elucidate the reason that this patient developed osteonecrosis of the trochlea. Haraldsson^9^ demonstrated that there are two vessels which enter the lateral humeral condyle posteriorly. These vessels take a long course through the lateral condylar ossification center all the way into the lateral portion of the trochlea. The trochlea itself receives its blood supply from these lateral vessels, as well as a separate vessel that penetrates the medial, nonarticular portion of the trochlea.^8^ These two blood supplies create a watershed area in the trochlear groove.^10^ This peculiar blood supply can be disrupted during the injury itself, during a closed or open reduction, or during internal fixation. One possible explanation for the osteonecrosis in this child is that the lateral vasculature was disrupted when the lateral condyle was fractured years earlier.

This patient was initially treated with cast immobilization. Given the minimal displacement, a closed reduction maneuver was not performed before casting. Other treatment options could have been closed or open reduction with internal fixation. Sullivan reports that type I or type II lateral condyle fractures with <2 mm displacement can be treated nonoperatively,^5^ such as in this patient. Others may opt for operative management in even minimally displaced lateral condyle fractures. Adequate reduction and fracture healing can likely be achieved either way, and both treatment options can be offered to the parents and the patient at presentation. Given the appropriate alignment and healing at the 3-month follow-up, we do not think that operative management would have prevented development of osteonecrosis in this patient.

Continued conservative management of this patient is in line with previous reports of those with the same pathology. In their systematic review in 2015, Claessen et al^1^ noted that all eight reported cases of Hegemann disease were treated conservatively with measures including rest and activity modification. Of the five patients with documented clinical outcomes, four of them had no pain after conservative management and the fifth patient had continued intermittent pain. We will continue to monitor his progress by both clinical symptoms and imaging.

This case report provides one of the most complete case examples, including late radiographs and MRI findings, or trochlear osteonecrosis after a nonsurgical lateral condyle fracture in a child. Physicians must be cognizant that pediatric distal humerus fractures can have this late complication, even when treated nonoperatively. Physicians should educate parents and patients to return to clinic, even years later, if they notice any symptoms such as clicking, pain, deformity, decreased range of motion, or decreased function. This rare complication should be identified early for the best possible outcome in these patients.
